# Hepatocellular Carcinoma With Portal Vein Tumor Thrombus Treated With Transarterial Chemoembolization and Sorafenib *vs.* ^125^Iodine Implantation

**DOI:** 10.3389/fonc.2021.806907

**Published:** 2021-12-23

**Authors:** Hong-Tao Hu, Jun-Peng Luo, Guang-Shao Cao, Zhen Li, Ming Jiang, Chen-Yang Guo, Hang Yuan, Quan-Jun Yao, Xiang Geng, Jung-Hoon Park, Hong-Tao Cheng, Li Jiang, Jun-Li Ma, Yan Zhao, Hai-Liang Li

**Affiliations:** ^1^ Department of Minimal-Invasive Intervention, The Affiliated Cancer Hospital of Zhengzhou University, Henan Cancer Hospital, Zhengzhou, China; ^2^ Department of Minimally Invasive Interventional Radiology, Sun Yat-sen University Cancer Center, State Key Laboratory of Oncology in South China, Collaborative Innovation Cancer for Cancer Medicine, Guangzhou, China; ^3^ Department of Intervention, Henan Provincial People’s Hospital, People’s Hospital of Zhengzhou University, Zhengzhou, China; ^4^ Department of Interventional Radiology, The First Affiliated Hospital of Zhengzhou University, Zhengzhou, China; ^5^ Department of Minimal-Invasive Intervention, Anyang Tumor Hospital/The Forth Affiliated Hospital of Henan University of Science and Technology, Anyang, China; ^6^ Biomedical Engineering Research Center, Asan Institute for Life Sciences, Asan Medical Center, Seoul, South Korea

**Keywords:** transarterial chemoembolization, hepatocellular carcinoma, portal vein tumor thrombosis, ^125^iodine implantation, prospective study

## Abstract

**Background and Aims:**

This study investigated the feasibility, safety, and efficacy of transarterial chemoembolization (TACE) combined with CT-guided ^125^iodine seed implantation for treatment of hepatocellular carcinoma (HCC) with first-branch portal vein tumor thrombosis (PVTT).

**Methods:**

This prospective, controlled, multicenter study included HCC patients with Barcelona Clinic Liver Cancer stage C disease and PVTT in the right and/or left portal veins. Patients were treated with either TACE and sorafenib or TACE and CT-guided ^125^iodine seed implantation and regularly evaluated for clinical response and adverse events, with treatment termination resulting from declining clinical status, loss to follow-up, or death.

**Results:**

This study demonstrated a significant between-group difference in median overall survival (OS); therefore, it was terminated early. A total of 123 patients were included in this study, with 52 patients in the TACE-sorafenib group and 71 patients in the TACE-^125^iodine group, without significant differences in baseline characteristics between groups. The median OS was 8.3 months (95% CI: 6.105–10.495) in the TACE-sorafenib group and 13.8 months (95% CI: 9.519–18.081) in the TACE-^125^iodine group. In a subgroup analysis of type IIa versus type IIb PVTT, the median OS was 17.5 months for type IIa and 7.1 months for IIb in the TACE-^125^iodine group. The median OS was 9.3 months for IIa and 4.0 months for IIb in the TACE-sorafenib group. Univariate and multivariate analyses confirmed that the PVTT type and treatment strategy were significant independent factors affecting OS. The objective response rates (ORR) for intrahepatic lesions and PVTT showed significant differences between groups. Most patients in both groups experienced minor adverse events related to TACE. The overall incidence of sorafenib-related adverse events or toxic effects was 90.4% in TACE-sorafenib group. In the TACE-^125^iodine group, the incidence of pneumothorax and minor hepatic subcapsular hemorrhage were 7.04% and 9.86%, respectively.

**Conclusions:**

This study showed that TACE-^125^iodine treatment significantly enhanced survival of patients with HCC and type II PVTT, especially subtype IIa, with minimal adverse events.

**Clinical Trial Registration:**

Chinese Clinical Trials Database, identifier ChiCTR-ONN-16007929.

## Introduction

In patients presenting with early-stage hepatocellular carcinoma (HCC), curative treatments are recommended, including transplantation, surgical resection, and ablation (1, 2). However, most patients with HCC are diagnosed at more moderate to advanced stages, with limited to no chance of receiving radical treatments. Portal vein tumor thrombosis (PVTT), a common complication in patients with advanced HCC, is observed in 64.7% of cases at autopsy and has a low median survival of 2.7–4.0 months who was untreated (3, 4). The poor prognosis of patients with PVTT is related to increased risks of tumor spread, bleeding from elevated portal blood pressures, and other complications, including ascites, jaundice, and liver failure due to decreased portal blood flow (5, 6). Many treatment strategies have been reported for HCC with PVTT, including transarterial therapy, ^90^Y radioembolization (^90^Y is expensive and cannot be used in China until the end of this research), external radiotherapy, systemic therapy, targeted therapy, and immunotherapy; however, none of these methods reached a consensus (5–11).

Sorafenib is the recommended first-line targeted therapy for patients with HCC and PVTT who have well-preserved liver function (12, 13). Unfortunately, clinical study had shown that the survival time of HCC patients with PVTT treated with sorafenib alone is limited (3, 14). Approximately 15% of patients abandon this treatment due to medication intolerance, while another 35% of patients require dose reductions. According to some studies, patients with PVTT who undergo treatment with sorafenib in combination with transarterial chemoembolization (TACE) demonstrate better survival, even in those with more advanced and recurrent disease (15–17). However, sorafenib and lenvatinib are used less frequently in real-world settings because of their high cost, medication intolerance, and unsatisfactory efficacy (15–18).

Yang et al. reported that TACE combined with endovascular implantation of a ^125^iodine seed strand was a feasible and effective treatment option in HCC patients with PVTT (19). Hu et al. reported a median OS of 13.1 months in a TACE-^125^iodine group versus 6.0 months in a TACE-alone group of HCC patients with PVTT in right or left portal vein branches (20). The advantages of implanting ^125^iodine seeds (20) in the PVTT are as follows: (a) The seeds continue to radiate the PVTT at a high dose and cause little damage to the surrounding normal tissues. (b) ^125^Iodine seed therapy is not affected by breathing exercises, with little loss of volume. (c) Compared with three-dimensional conformal radiotherapy, ^125^iodine seeds have better biological effects, and can shorten the length of hospital stay and reduce costs. These prior studies were all retrospective in design though; thus, prospective, controlled studies are necessary to compare the efficacy of standard sorafenib treatment versus ^125^iodine seed implantation in patients with advanced liver cancer.

Therefore, this prospective, multicenter clinical trial aimed to investigate the feasibility, safety, and efficacy of TACE combined with CT-guided ^125^iodine seed implantation versus TACE combined with sorafenib for treatment of patients with HCC and first-branch portal vein tumor thrombosis (type II).

## Materials and Methods

### Study Design

Based on a previous report (20), we hypothesized a median overall survival time of 7 months for patients treated with TACE and sorafenib and a median overall survival time of 14 months for patients treated with TACE combined with CT-guided ^125^iodine seed implantation. For an alpha level of 0.05 (one-sided test), the total required sample size (distribution ratio of 1:2) was 180 to obtain a 90% power without patient loss or switching groups (PASS, log-rank test, median survival time). Because of the nature of the treatment and its associated side effects, double-blinding techniques were not utilized. We planned involved all of 180 patients in two years (60 patients in TACE-sorafenib group and 120 patients in TACE-^125^iodine group). This study’s primary endpoint was overall survival time, the secondary endpoint was tumor response, and additional endpoints included procedure-related complications and adverse events.

**Figure 1:**
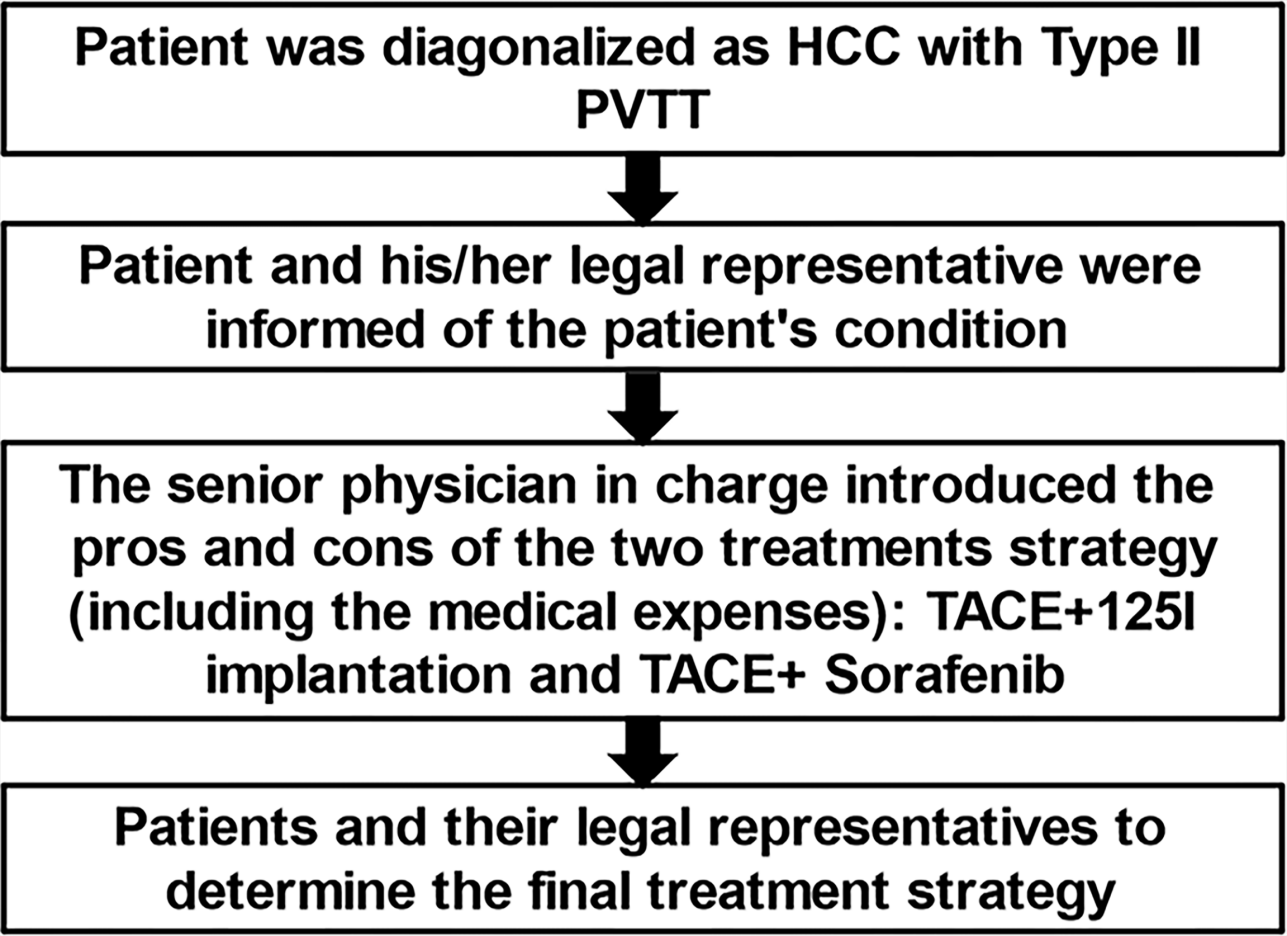
Doctor-patient decision-making model flowchart.

This study was approved by the Ethics Committee of our institution and was registered in the Chinese Clinical Trials Database (number ChiCTR-ONN-16007929). Before undergoing treatment, patients were informed of the risks, benefits, and costs of TACE-sorafenib versus TACE-^125^iodine treatment strategies by the attending physicians (QJY and XG), and we adopt a joint doctor-patient decision-making model (**Figure 1**), the patients choose one of two treatments. All patients were treated at four medical centers in Henan province, and all researchers were trained in this study. All patients provided written informed consent for participation in this study.

### Patient Selection and Enrollment Criteria

HCC was diagnosed based on the criteria of the European Association for the Study of Liver Disease/American Association for the Study of Liver Disease (21). The inclusion criteria for the study population were as follows: Eastern Cooperative Oncology Group (ECOG) performance status of 0–2; Child-Pugh class A or B (score ≤ 8), and Barcelona Clinic Liver Cancer stage C (presence of PVTT in the right and/or left portal veins on CT images). Patients were excluded if this was not their initial diagnosis of HCC, or if they had previously undergone other surgical procedures, ablation treatments, sorafenib therapy, systemic chemotherapy, intra-arterial chemoinfusion, or TACE. Initiation and follow-up of treatment were carried out at one of four centers, with baseline dynamic contrast-enhanced computed tomography (CT) imaging obtained within 7 days before treatment. PVTT was confirmed by enhanced CT or enhanced Magnetic Resonance Imaging (MRI), the imaging features of PVTT included solid lesions within the portal vein in all the phases of intravenous enhanced 3-phase computed tomography, especially with an enhancement of contrast in the arterial phase and washout in the portal venous phase of the procedure (22).

### Classification of PVTT

We classified PVTTs into three subgroups based on Cheng’s classification (23), as follows: (a) type III was defined as a PVTT in the main portal vein; (b) type II was divided into IIa and IIb subtypes, with type IIa defined as a PVTT in a first-order portal vein branch (the right or left portal vein only) and type IIb defined as a PVTT in both the left and right first-order portal vein branches; and (c) type I defined as a PVTT in a second- or lower-order portal vein branch. All eligible patients with a type II PVTT (IIa or IIb) were included in this study.

### TACE Procedure

As has previously been reported (22), TACE was performed by administering doxorubicin mixed with 5–20 mL lipiodol (Lipiodol Ultrafluide, Laboratoire Guerbet, France) to both groups. The dose of doxorubicin was 50–75 mg/m^2^ (Haizheng Pharmaceutical Co. Ltd., China) adjusted based on patient liver function, tumor size, vascularity, presence of an arterioportal shunt, and body surface area. All tumor-feeding arteries were superselected by a microcatheter (Terumo, Tokyo, Japan), and the mixture was injected at a rate of 1 mL/min until stagnation of blood flow was observed under fluoroscopic guidance. Gelatin sponge particles with a 500–700-um size was used to block feeding arteries of tumors. If an artery–portal vein shunt was performed, it was occluded before mixture embolization by big-size polyvinyl alcohol (Polyvinyl Alcohol Foam Embolization Particles; Cook Medical Inc., Bloomington, IN, USA) or a spiral steel ring based on angiography images and the doctor’s experience.

### Sorafenib Treatment

All patients in the TACE-sorafenib group were started on oral sorafenib (400 mg, bid) at 3–7 days before the first TACE session, and the patients stopped sorafenib for 3-7 days during TACE. Sorafenib dose reductions were based on toxicities.

### ^125^Iodine Seed Implantation Procedure

**Figure 2:**
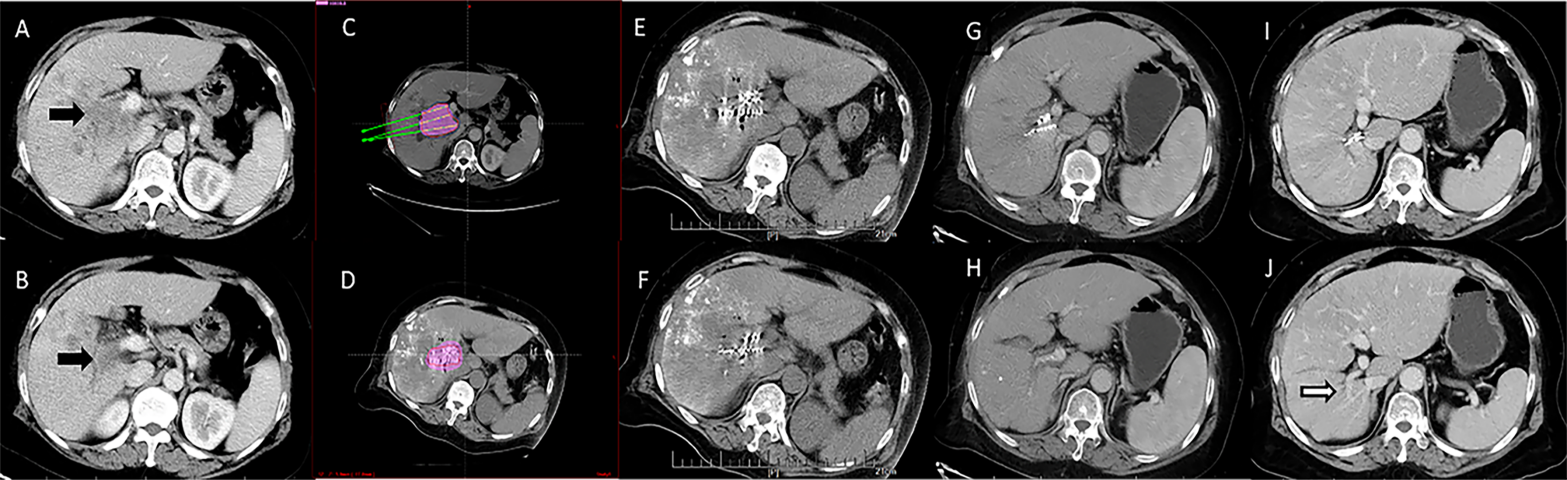
Computed tomography imaging of a 67-year-old woman with hepatocellular carcinoma complicated by portal vein tumor thrombosis in the right branch (Type IIa). **(A, B)** Enhanced computed tomography imaging showing a portal vein tumor thrombus originating from the right branch of the portal vein. The image before iodine seed implantation showed **(C)** The spatial distribution of seeds according to the treatment planning system (TPS), and **(D)** dosage verification, with good results according to the TPS. **(E, F)** Computed tomography imaging demonstrated that the iodine seed was distributed in the PVTT, the PVTT had decreased in size, and **(G, H)** the portal vein was partly recanalized at 3 months after seed implantation. **(I, J)** The portal vein was completely recanalized at 12 months after the procedure, and the patient remained alive until the end of follow-up.

**Figure 3:**
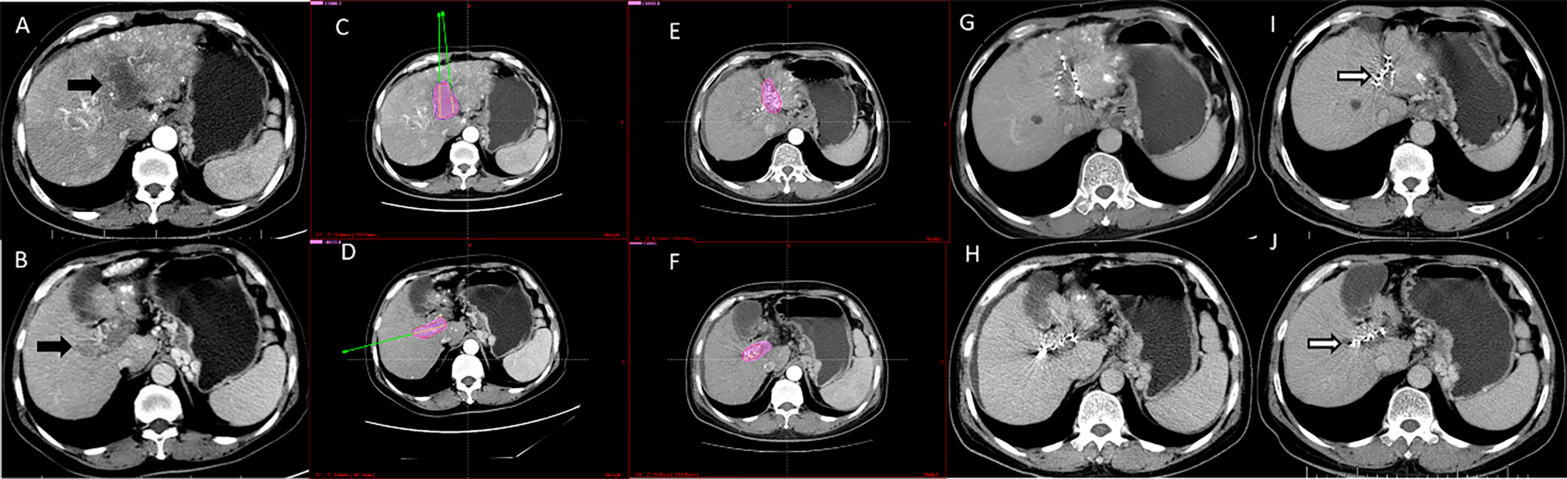
Computed tomography imaging of a 56-year-old man with hepatocellular carcinoma complicated by PVTT (Type IIb). **(A, B)** Enhanced computed tomography imaging showed that the thrombus had originated from the left and right branches of the portal vein. **(C, D)** The image prior to iodine seed implantation showed the dosage distribution of seeds in the left and right branches according to the TPS. **(E, F)** The iodine seed implantation was performed in left and right branches during one procedure, with the dosage verification image showing iodine seed implantation according to the treatment planning system. **(G, H)** Computed tomography imaging demonstrated that the PVTT had decreased in size, but the portal vein still showed obstruction at 3 months after seed implantation. **(I, J)** Twelve months after the procedure, the PVTT had shrunk more; however, there were no signs of recanalization. This patient died due to liver failure at 16 months after the procedure.

Four days after the first TACE session, patients in the TACE-^125^iodine group underwent repeat testing of liver and coagulation function. If liver and coagulation function had recovered to Child-Pugh Class A and normal, respectively, CT-guided ^125^iodine implantation was performed, as has previously been described (19). If the patient has liver function Child-Pugh B or abnormal blood coagulation test after TACE, we will continue to correct liver function and coagulation function before implanting seeds, and complete all corrections in a short time, and successfully carry out seed implantation treatment. We used a treatment planning system (TPS) (FTT Technology Ltd. Co, Beijing, China) to calculate the formula dosage, number, spatial distribution, intensity of radioactivity, and matched peripheral dosage of seeds before implantation. The ^125^iodine seeds were then implanted into the cancerous embolus under CT guidance 5 mm apart along the length and 10 mm apart along the width (**Figures 2**, **3**). After this procedure, all patients received conventional liver protection treatment.

### Follow-Up and Repeated TACE or ^125^Iodine Implantations

Patients underwent follow-up evaluations at 4-week intervals after TACE, including detailed history and physical examinations, laboratory testing, and abdominal contrast CT or magnetic resonance (MR) imaging. Laboratory tests included a complete blood cell count, liver function, coagulation function, α-fetoprotein, and creatinine level.

TACE was repeated depending on the radiological response. When new lesions or residual viable tumors were observed in patients with a liver function of Child-Pugh Class A or B, repeated TACE procedures were performed. In the TACE- ^125^iodine group, residual viable tumors or recurrent PVTTs were identified at 2 months after seed implantation, with repeat performance of ^125^iodine seed implantation in these cases. Treatment was terminated if patients were unable to tolerate the therapy, as evidenced by declining clinical status, loss to follow-up, or death.

### Patient Assessments

The end of the follow-up period was determined to be either at death or on 31 December, 2019. We compared the median overall survival (OS) between treatment groups, and OS was defined as the time from the enrollment until death or the last follow-up visit.

All data, including clinical, laboratory, and radiologic records, were obtained from the electronic medical records of the four hospitals and reviewed. Adverse events were reported according to the National Cancer Institute Common Terminology Criteria for Adverse Events, version 4.0 (24). In the TACE-^125^iodine group, ^125^iodine seed implantation-related adverse events were evaluated at 7 days after seed implantation. Sorafenib-related adverse events were monitored until discontinuation of the oral medication. Since symptoms of post-embolization syndrome (i.e., abdominal pain, fever without an infectious focus, nausea, and vomiting) were expected, they were not documented separately.

The intraparenchymal lesion response rate was evaluated based on the modified Response Evaluation Criteria in Solid Tumors (mRECIST) assessment for HCC at the last follow-up by two experienced radiologists (HLL and CYG) (25). Lesions were determined to demonstrate either a complete response (CR), partial response (PR), stable disease (SD), or progressive disease (PD).

To evaluate PVTT response, the system reported by Yang et al. was used, described as follows (19): CR = PVTT disappearance and restoration of portal vein blood flow; PR = reduction in a PVTT by > 30% in the greatest cross-sectional area or portal vein blood flow improvement; SD = reduction in a PVTT by < 30% in the greatest cross-sectional area or unchanged, and PD = PVTT extended.

### Statistical Analyses

All statistical analyses were performed using SPSS version 22.0 (SPSS, Chicago, IL, USA). To determine significant differences between groups, the Student’s *t*-test, chi-squared test, or Fisher’s exact test were used. Kaplan-Meier survival curves were constructed and evaluated for both groups. Univariate analysis was performed using the log-rank test, and multivariate analysis was conducted using a Cox proportional-hazards regression model on variables with *P*-values of < 0.10 in the univariate analyses. Two-tailed statistical tests were performed, and *P*-values of < 0.05 were considered significant.

## Results

### Declaration of Study Termination

From January 2017 to December 2018, a total of 127 HCC patients with first-branch PVTTs were included in this study. Although not included in the estimated number of cases in two years. There was a significant difference in the median OS of patients in the two treatment groups by statistical analysis; therefore, the study was terminated early after discussion with researchers and approval by the Ethics Committee of our institution.

### Patient Characteristics

In the TACE-sorafenib group three patients were lost to follow-up, and one patient quit the study for other treatments in the TACE-^125^iodine group. Based on patient request, the 123 patients were divided into either a TACE-sorafenib group (n = 52), with a median age of 61.5 years (range, 34–78 years), or a TACE-^125^iodine group (n = 71), with a median age of 57 years (range, 33–85 years). Detailed baseline characteristics, including sex, age, ECOG score, Child-Pugh score, tumor number, largest tumor diameter, total tumor diameter, type of PVTT (IIa or IIb), AFP level, and blood test results are shown in **Table 1**. Baseline characteristics between the TACE-sorafenib group and the TACE-^125^iodine group were not statistically different.

**Table 1:** Baseline characteristics of patients.

Variable	TACE-^125^iodine group (n = 71)	TACE-sorafenib group (n = 52)	*χ^2^* (*t*)	*P*-value
Sex (M/F)	64/7	45/7	0.386	0.534
Age (≤60 year/>60 year)	44/27	25/27	2.353	0.125
Median age (year)	57	61.5	-0.892	0.774
ECOG (0/1-2)	29/42	25/27	0.637	0.425
Child-Pugh classification (A/B)	52/19	40/12	0.216	0.642
Child-Pugh score†	5.83 ± 1.12	5.942 ± 1.07	-0.554	0.261
Tumor number (1/≥2)	34/37	22/30	0.377	0.539
Maximum diameter (<60 mm/≥60 mm)	48/23	36/16	0.037	0.848
Total diameter (<100mm/≥100 mm)	51/20	41/11	0.784	0.376
Tumor distribution (Right or Left/Bilobar)	29/42	14/38	2.559	0.110
Classification of PVTT (IIa/IIb)	35/36	30/22	0.849	0.357
Preoperative AFP (≥/< 400 ng/mL)	46/25	32/20	0.137	0.712
TBL (umol/L)†	21.30 ± 1.82	20.57 ± 1.89	0.275	0.784
Albumin (g/L)†	38.76 ± 0.65	38.12 ± 0.59	0.692	0.490
PT (s) †	13.19 ± 0.20	13.20 ± 0. 19	-0.025	0.98
WBC (×10^9^/L)†	5.41 ± 0.27	5.26 ± 0.24	0.386	0.700
RBC (×10^12^/L)†	4.33 ± 0.07	4.13 ± 0.07	1.828	0.070
HGB (g/L) †	133.01 ± 2.24	129.83 ± 2.04	1.010	0.314

Unless otherwise indicated, data are presented as numbers of patients.

†Data are presented as mean (standard deviation).

AFP, alpha fetoprotein; ECOG, Eastern Cooperative Oncology Group; HGB, hemoglobin; PT, prothrombin time; PVTT, portal vein tumor thrombosis; RBC, red blood cell; TACE, transarterial chemoembolization; TBL, total bilirubin; WBC, white blood cell.

### Follow-Up and Overall Survival Time

**Figure 4:**
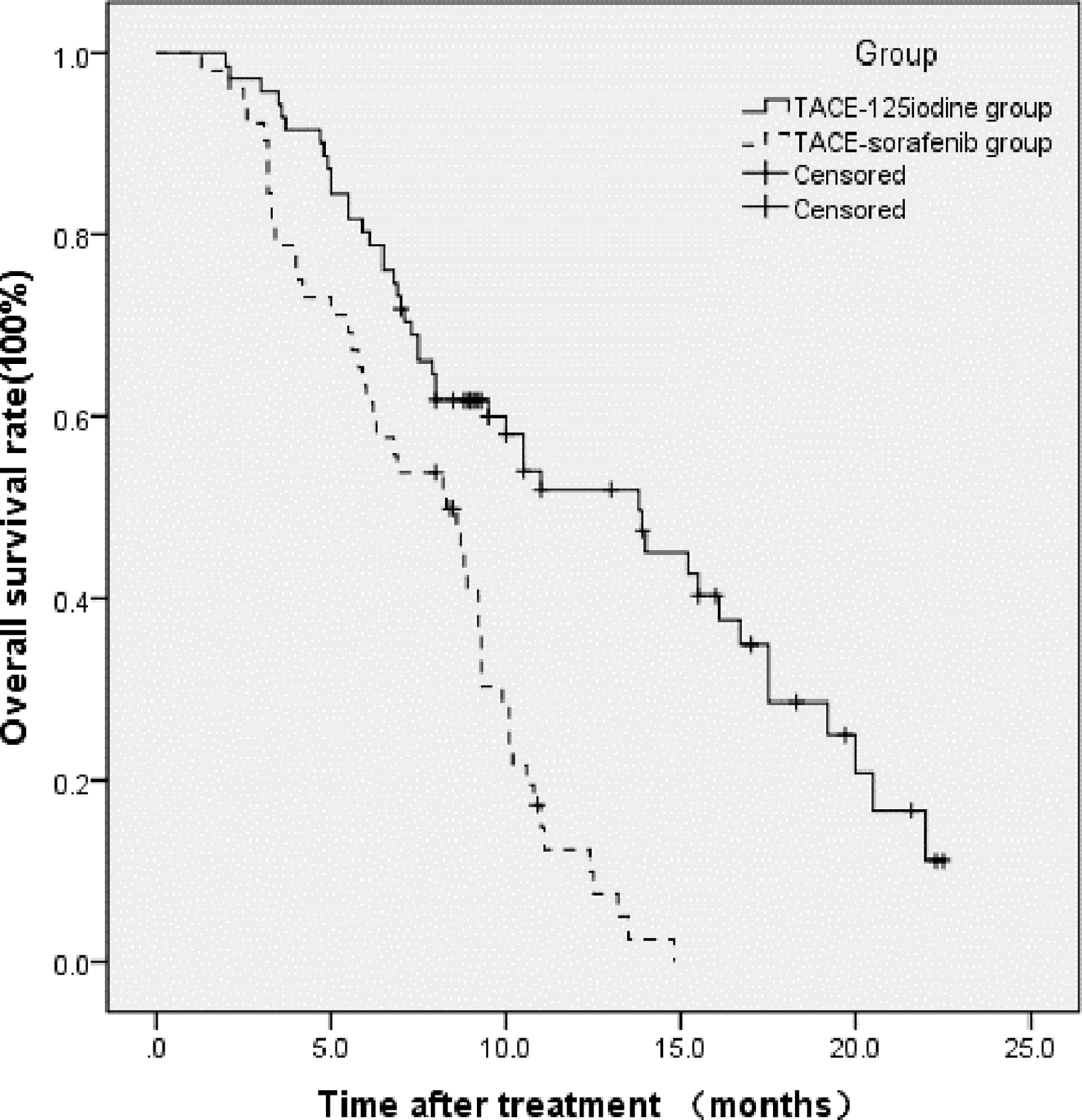
Overall survival in the two groups (*P* < 0.001, log-rank test).

This study’s follow-up period ended in December of 2019. At the 6-month postoperative follow-up, 32 patients (32/52, 61.5%) in the TACE-sorafenib group and 57 patients (57/71, 80.3%) in the TACE-^125^iodine group had survived. At 12-month and 18-month follow-ups, 7 and 0 patients had survived in the TACE-sorafenib group, and 24 and 9 patients had survived in the TACE-^125^iodine group, respectively. The 6-month, 12-month, and 18-month survival rates were 80.3%, 51.9%, and 28.6%, respectively, in the TACE-^125^iodine group and 61.5%, 12.4%, and 0%, respectively, in the TACE-sorafenib group. The median OS was 8.3 months (95% CI: 6.105–10.495) in the TACE-sorafenib group and 13.8 months (95% CI: 9.519–18.081) in the TACE-^125^iodine group (*χ* ^2^ = 24.204, *P* < 0.001). **Figure 4** shows the patient survival curve.

In the subgroup analysis of type IIa versus type IIb PVTT, the median OS was 17.5 months (95%CI: 15.66–19.33) for type IIa and 7.1 months (95% CI: 6.25.–7.95) for type IIb in the TACE-^125^iodine group. In the TACE-sorafenib group, the median OS was 9.3 months (95% CI: 15.66–19.33) for type IIa and 4.0 months (95% CI: 6.25–7.95) for type IIb (**Table 2**).

**Table 2:** Subgroup analysis of overall survival in type IIa versus type IIb portal vein tumor thromboses.

	Type IIa (mOS, 95% CI)	Type IIb (mOS, 95% CI)	*χ* ^2^	*P*-value
TACE-^125^iodine group (months)	17.5 (15.66-19.33)	7.1 (6.25- 7.95)	24.303	<0.001
TACE-sorafenib group (months)	9.3 (8.92-9.68)	4.0 (2.16- 5.84)	8.509	0.004
*χ* ^2^	31.288	6.636		
*P*-value	<0.001	0.010		

mOS, median overall survival; TACE, transarterial chemoembolization.

### Factors Associated With Patient Survival Analysis

The following variables were entered into the univariate analysis: gender, age, ECOG score, Child-Pugh classification, liver tumor number and size, liver tumor location, PVTT type (IIa or IIb), preoperative AFP, and treatment strategy. Univariate analysis results revealed that the PVTT type (IIa or IIb) and the treatment strategy (TACE combined with sorafenib versus TACE combined with ^125^iodine) were significant independent factors associated with patient prognosis, and the multivariate analysis confirmed these variables were significant independent factors for OS (**Table 3**).

**Table 3:** Univariate and multivariate analyses of prognostic factors for overall survival.

	Univariate analysis			Multivariate analysis		
Variable	n	*χ* ^2^ (log-rank)	*p*	RR	95% CI	*p*
Sex (M/F)	109/14	0.900	0.343			
Age (≤60 year/>60 year)	69/54	0.359	0.549			
ECOG (0/1-2)	54/69	3.162	0.075			
Child-Pugh (A/B)	92/31	0.729	0.393			
Tumor number (1/≥2)	56/67	0.063	0.802			
Maximum diameter (<60 mm/≥60 mm)	84/39	0.076	0.783			
Total diameter (<100 mm/≥100 mm)	92/31	0.476	0.490			
Classification of PVTT (Type IIa/Type IIb)	65/58	30.409	<0.001	3.465	(2.247, 5.344)	<0.001
Preoperative AFP (≥/<400 ng/mL)	78/45	0.288	0.591			
Tumor distribution (Right or Left/Bilobar)	43/80	0.217	0.642			
Treatment (TACE-^125^iodine/TACE-sorafenib)	71/52	24.204	<0.001	3.224	(2.050, 5.069)	<0.001

AFP, alpha fetoprotein; ECOG, Eastern Cooperative Oncology Group; PVTT, portal vein tumor thrombosis; RR, relative risk; TACE, transarterial chemoembolization.

### Intrahepatic Lesions and PVTT Response

The ORR of intrahepatic lesions was 58.3% for the TACE-sorafenib group and 81.6% for the TACE-^125^iodine group, with a significant difference between groups (*χ* ^2^ = 8.579, *P* = 0.035). The ORR for PVTTs was 43.8% for the TACE-sorafenib group and 75.4% for the TACE-^125^iodine group (**Table 4**), with a significant different between groups (χ^2^ = 14.365, *P* = 0.002).

**Table 4:** The objective response rate of intrahepatic lesions and portal vein tumor thromboses.

	Intrahepatic lesions		PVTT	
	TACE-^125^iodine group	TACE-sorafenib group	TACE-^125^iodine group	TACE-sorafenib group
CR	4	1	6	0
PR	52	27	46	21
SD	11	14	12	18
PD	2	6	5	9
ORR	81.6%	58.3%	75.4%	43.8%

Unless otherwise indicated, data are numbers of patients.

CR, complete response; ORR, objective response rate; PD, progressive disease; PR, partial response; PVTT, portal vein tumor thrombosis; SD, stable disease; TACE, transarterial chemoembolization.

### Repeated TACE and ^125^Iodine Seed Implantation Procedures

In the TACE-sorafenib group, the number of TACE procedures ranged from 1 to 9, with an average of 4.535 ± 2.41. The sorafenib treatment duration ranged from 1.3–14.8 months. The average number of TACE procedures was 5.077 ± 2.96 (range, 1–8) in the TACE-^125^iodine group, with an average number of ^125^iodine seed implantations of 1.4 ± 0.5 (range, 1–2). There was no statistically significant difference (*t* = -1.118, *P* = 0.073) in the average number of TACE procedures between the two groups. The number of implanted ^125^iodine seeds ranged from 4 to 19 (radiation dose from 2810-223690 cGy), with an average of 7 ± 1 seeds (radiation dose 26901.24 ± 3480.68 cGy).

### Child-Pugh Score Changes After 3 Months of Treatment

Patients who survived for less than 3 months were not included in this specific analysis. Child-Pugh scores before and after treatment were 5.77 ± 1.06 and 5.39 ± 0.67 in the TACE-^125^iodine group (*t* = 2.578, *P* = 0.012), respectively, and 5.90 ± 1.08 and 6.02 ± 0.96 in the TACE-sorafenib group (*t* = -0.643, *P* = 0.523), respectively. Child-Pugh scores had significantly improved at 3 months after initial treatment in the TACE-^125^iodine group without a significant change in the TACE-sorafenib group. In addition, the Child-Pugh score of the TACE-^125^iodine group was significantly lower than the TACE-sorafenib group (*t* = -4.192, *P* < 0.001).

### Causes of Patient Death During Follow-Up

During the follow-up period, 48 of the 52 patients (92.3%) in the TACE-sorafenib group and 46 of the 71 patients (64.8%) in the TACE-^125^iodine group died. The causes of death in the TACE-sorafenib group were liver failure (19 cases), gastrointestinal bleeding (15 cases), and tumor progression (14 cases). In the TACE-^125^iodine group, the causes of death were tumor progression (25 cases), gastrointestinal bleeding (11 cases), and liver failure (10 cases). There were statistically significant differences in the causes of death between the two groups of patients (*χ* ^2^ = 6.471, *P* = 0.039) (**Table 5**).

**Table 5:** Cause-of-death analysis.

	TACE-^125^iodine group	TACE-sorafenib group	*χ* ^2^	*P*-value
Gastrointestinal bleeding	11	15	6.471	0.039
Liver failure	10	19		
Tumor progression	25	14		

TACE, transarterial chemoembolization.

### Adverse Events Related to TACE

Minor TACE-related adverse events, including liver dysfunction, fever, abdominal pain, nausea, and vomiting, were reported in most patients in both groups. These adverse events were treated symptomatically in the hospital, and all patients recovered from these events within 7 days. Severe adverse events related to TACE were not encountered during or after the TACE procedure in any patients.

### Adverse Events Related to Sorafenib

The overall incidence of adverse events or toxic effects related to sorafenib use was 90.4% (47/52). The most common minor toxic effects included diarrhea (73.1%, 38/52), hand-foot skin reactions (67.3%, 35/52), rashes (40.4%, 21/52), fatigue (34.6%, 18/52), and grade I myelosuppression (21.2%, 11/52). The most common grade 3 to 4 adverse events were diarrhea (7.7%, 4/52), hand-foot skin reactions (5.8%, 3/52), and rashes (5.8%, 3/52). For these adverse events, the following strategies were implemented as needed: reducing sorafenib dosage (changing from 400 mg bid to 400 mg qd), discontinuation of sorafenib, or symptomatic treatment. If adverse events decreased to grade 1-2, the medication was continued until the normal dosage was restored. No sorafenib-related deaths occurred.

### Adverse Events Related to ^125^Iodine Seed Implantation

Few acute adverse events were reported related to ^125^iodine seed implantation. Five patients (5/71) developed pneumothoraxes and collapsed lungs (< 30% of the ipsilateral lung) with recovery after conservative treatment. In seven (7/71) cases, minor subcapsular liver hemorrhages were observed on CT imaging after finishing the procedure; however, these hemorrhages were treated conservatively with success. In three cases, seed flotation to the normal liver parenchyma was observed but did not require medical management. Other potentially severe adverse events were not observed in relation to^125^iodine seed implantation.

## Discussion

In the present study, we compared the outcomes of patients with advanced HCC and type II PVTT treated with either TACE combined with sorafenib or TACE combined with CT-guided ^125^iodine seed implantation as a first-line therapy. We found that the median OS was significantly higher in the TACE-^125^iodine group than in the TACE-sorafenib group. Furthermore, ORRs were significantly higher for both liver parenchymal lesions and PVTTs in the TACE-^125^iodine group than in the TACE-sorafenib group. Furthermore, univariate and multivariate analyses demonstrated that the PVTT subtype and the treatment strategy were significant independent factors related to patient OS. To our knowledge, this clinical study was the first prospective, multicenter study to compare outcomes of TACE combined with sorafenib versus TACE combined with ^125^iodine seed implantation for treatment of patients with advanced HCC and type II PVTT.

An increasing number of treatment strategies and drugs have been developed for palliation and survival benefit in patients with PVTT. Sorafenib and other related drugs, including regorafenib, brivanib, and nivolumab, have shown promising preliminary results as both first-line and second-line systemic therapies for advanced-stage HCC (6, 26, 27). Unfortunately, the impact of these agents on clinical outcomes and their relative merits in patients with PVTT are yet to be fully understood. In addition, their high prices make them virtually impossible to obtain for most HCC patients in China.

On the other hand, some doctors have focused on more localized treatment strategies for HCC patients with PVTT. For example, Song et al. performed hepatic arterial infusion chemotherapy in patients with HCC and PVTT, with a median OS of 7.1 months (28). Abouchaleh reported a median OS of 13.3 months using the ^90^Y radioembolization method for patients with HCC and PVTT (10). Furthermore, a median OS of 10.2 months and a response rate of 52% was reported after external radiotherapy in this patient population (11). A meta-analysis showed that the 1-year survival rates for the 3-dimensional conformal radiotherapy (3DCRT), selective internal radiotherapy (SIRT) and stereotactic body radiotherapy (SBRT) groups were 43.8%, 46.5% and 48.5% (p = 0.635). The difference between these groups was not statistically significant (p = 0.635) (29). ^125^Iodine seed implantation is used as internal radiation therapy, which is as effective as SBRT and 3DCR. A randomized, open-label clinical trial to evaluate the efficacy and safety of TACE combined with radiotherapy and sorafenib in the treatment of hepatocellular carcinoma and gross vascular invasion (30). The results show that TACE combined with radiotherapy can prolong the progression-free survival and OS of patients compared with sorafenib. Also, a median OS of 13.1 months has been achieved with use of TACE combined with brachytherapy (18). In our prospective, multicenter clinical study, the median OS was 13.8 months in the TACE-^125^iodine group. Therefore, this prospective study achieved almost the same median OS as this previous study and proved the efficacy of brachytherapy in PVTT. Due to the inherent strengths of prospective and multicenter studies, our conclusions are likely more convincing.

Interestingly, after we analyzed differences in subtype IIa versus subtype IIb PVTTs, we found that when a PVTT involved only the left or the right branch (subtype IIa), the median OS was significantly higher than when a PVTT involved both the left and right branches (subtype IIb) in the TACE-^125^iodine (17.5 *vs*. 7.1 months) and TACE-sorafenib (9.1 *vs*. 4 months) groups. This finding can be explained because blockage of only one portal vein branch can preserve more liver function and can maintain relatively low portal pressures. From a biological perspective, tumors that simultaneously invade both the left and right portal vein branches (subtype IIb) have a higher degree of malignancy and a worse treatment effect than subtype IIa PVTTs. From another perspective, patients with better liver function reserves and lower portal pressures can also tolerate more treatments and can obtain better survival benefits.

After careful investigation of treatment efficacy in subtype II PVTTs, we determined that the hazard and prognosis for subtype IIb PVTTs are equivalent to those of main PVTTs; however, the staging is better. Zhu et al. reported that the median OS of patients with type III PVTTs (a main portal vein thrombosis) was 3.0 months in the TACE-sorafenib group (30). This result is similar to our findings at 4 months for subtype IIb (portal vein thrombosis) PVTTs in the TACE-sorafenib group. Even though the median OS was significantly shorter for subtype IIb PVTTs than subtype IIa PVTTs (7.1 *vs*. 17.5 months, respectively) in the TACE-^125^iodine group, the 7.1-month OS was still superior to subtype IIb patients in the TACE-sorafenib group. Yang et al. described a patient with a main PVTT who achieved a median OS of 154.0 ± 11.2 days (19), similar to our results for subtype IIb PVTTs in the TACE-^125^iodine group by using TACE combined with endovascular implantation of a ^125^iodine seed strand. This finding is interesting because previous studies have repeatedly shown that patients with branch PVTTs have a significantly longer survival time than those with main PVTTs (14, 31). We agree that patients with subtype IIa PVTTs have a better prognosis than patients with type III PVTTs; however, subtype IIb PVTTs have a similar prognosis to type III PVTTs.

We analyzed the liver function of patients at 3 months after treatment. We found that the Child-Pugh score decreased significantly in the TACE-^125^iodine group (*P* = 0.012) without a significant change in the TACE-sorafenib group (*P* = 0.523). One possible reason for this improvement in liver function is that PVTTs shrank rapidly after ^125^iodine seed implantation and that portions of the portal vein were recanalized, leading to lower portal vein pressures, increased portal blood supply to the liver, and restoration of liver function. HCC treatment can be considered a delicate balance between adequately treating tumors and maximally preserving remaining liver function. The extra burden that PVTTs impose on liver cancer patients fundamentally challenges the “dual blood supply” principle underlying transarterial embolization. Reductions in PVTTs and restoration of liver function therefore provides patients with more treatment opportunities.

We also found that the average number of TACE procedures were not significantly different between the two groups, which implies that patients in the TACE-^125^iodine group underwent less TACE procedures than the TACE-sorafenib group over the same time period, which may also have preserved liver function on the contralateral side and improved patient survival.

We found that the leading causes of death during follow-up were significantly different between the two groups. The most common cause of death was liver failure in the TACE-sorafenib group and tumor progression in the TACE-^125^iodine group. This finding supports the theory mentioned above that seed implantation could quickly improve liver function in patients. On the other hand, this result also implies that ^125^iodine seed implantation is a local therapy and does not have a therapeutic effect on liver parenchymal tumors, despite PVTT control reducing intrahepatic tumor spread.

In contrast, although sorafenib had a low ORR for PVTTs compared with ^125^iodine seed implantation (43.8% *vs*. 75.4%), it may have a concurrent therapeutic role for both liver tumors and PVTT, which is an advantage of this treatment strategy. A meta-analysis shows that radiotherapy combined with sorafenib is an option for advanced hepatocellular carcinoma (32). Therefore, we hypothesize that TACE combined with ^125^iodine seed implantation and sorafenib treatment may avoid the disadvantages of these two treatment modalities and may strengthen control of both intrahepatic tumors and PVTTs in order to achieve better curative effects. Of course, this hypothesis requires further exploration in future works.

Univariate and multivariate analyses showed that the PVTT subtype and the treatment strategy were independent pretreatment prognostic factors for OS. This finding implies that TACE combined with ^125^iodine seed implantation may be an effective and economical treatment option for HCC patients with type II PVTT. In addition, prognostic differences for the various subtypes of PVTT must be considered when choosing appropriate therapeutic strategies for patients.

The most common adverse events of CT-guided ^125^iodine implantation included puncture site pain, pneumothorax, seed transmigration, and slight subcapsular hemorrhages, which were well tolerated with full patient recovery. No major complications were observed in this study, as has previously been reported.

There were several limitations to the current study. First, the sample size was small, thereby introducing inherent bias. Thus, larger sample study was necessary to evaluate the efficacy of TACE-^125^iodine treatment in HCC patients with PVTT. Second, although we used the TPS planning system, the dose distribution of seeds may have been uneven because seed implantation was performed under local anesthesia without a three-dimensional printing template, potentially creating differences in the treatment effect. Our next research direction will focus on how to ensure a more uniform particle dose distribution for PVTT. Although we terminated the experiment early, there may be a problem of small sample size, but the results have shown significant statistical differences. Therefore, termination of the experiment is the best choice from the perspective of patient benefit and ethics.

In conclusion, our study showed that TACE-^125^iodine treatment could significantly enhance survival of HCC patients with type II PVTT. This strategy is more effective and affordable than sorafenib for treatment of PVTT, especially for subtype IIa, with minimal adverse events.

## Data Availability Statement

The original contributions presented in the study are included in the article/supplementary material. Further inquiries can be directed to the corresponding author.

## Ethics Statement

The studies involving human participants were reviewed and approved by Ethics Committee of the Affiliated Cancer Hospital of Zhengzhou University review board. The patients/participants provided their written informed consent to participate in this study.

## Author Contributions

Conception and design: H-LL. Patient selection and treatment: HTH, J-PL, G-SC, ZL, MJ, LJ, J-LM, and YZ. Data collection, analysis, and interpretation: H-TH, J-PL, G-SC, ZL, MJ, C-YG, HY, Q-JY, XG, H-TC, LJ, J-LM, and YZ. Data interpretation: H-LL, H-TH, J-PL, G-SC, ZL, and MJ. Undertook steering committee activities and critical statistical processing: LJ, J-LM, and YZ. Manuscript writing: H-LL, H-TH, H-TC, and J-PL. Manuscript reviewing: J-HP. All authors contributed to the article and approved the submitted version.

## Funding

This study was funded by Guerbet France, Henan Province Natural Science Foundation (212300410403), Henan Province Medical Science and Technology Research Project (201701032) and National Science and Technology Major Project of the Ministry of Science and Technology of China (2018ZX10303502).

## Conflict of Interest

The authors declare that the research was conducted in the absence of any commercial or financial relationships that could be construed as a potential conflict of interest.

## Publisher’s Note

All claims expressed in this article are solely those of the authors and do not necessarily represent those of their affiliated organizations, or those of the publisher, the editors and the reviewers. Any product that may be evaluated in this article, or claim that may be made by its manufacturer, is not guaranteed or endorsed by the publisher.
